# Social Facilitation in Fear Appeals Creates Positive Affect but Inhibits Healthy Eating Intentions

**DOI:** 10.3389/fpsyg.2022.838471

**Published:** 2022-03-03

**Authors:** Rachel L. Bailey, Tianjiao Grace Wang, Jiawei Liu, Russell B. Clayton, Kyeongwon Kwon, Vaibhav Diwanji, Farzaneh Karimkhanashtiyani

**Affiliations:** ^1^School of Communication, Florida State University, Tallahassee, FL, United States; ^2^Department of Communication, Bradley University, Peoria, IL, United States; ^3^School of Journalism and Communication, Jinan University, Guangzhou, China; ^4^William Allen White School of Journalism and Mass Communication, University of Kansas, Lawrence, KS, United States

**Keywords:** cue reactivity, fear appeals, social facilitation of eating, health communication, coactivation

## Abstract

The social facilitation of eating plays a significant role in influencing individuals’ eating decisions. However, how social eating cues are processed in health promotion messages is unclear. This study examined individuals’ food craving in response to social cues in images (Experiment 1) and emotional experiences, perceived threat, perceived efficacy, behavioral intentions, and motivational coactivation elicited by social eating cues in obesity prevention fear appeals (Experiment 2). Results suggested that the presence of a group of people eating in an image facilitated food craving for the presented foods. Moreover, fear appeals that presented obesity and its consequences with more social eating cues, versus individual eating cues, generated greater positive emotional responses, perceived threat severity, response and self-efficacy, and motivational coactivation indicating more attention and threat vigilance. However, these cues also generated fewer self-reported intentions to change unhealthy eating behaviors. Implications and suggestions for future research are discussed.

## Introduction

The use of fear appeals to limit unhealthy behaviors is contentious. Decades of research have revealed mixed findings regarding their efficacy, and meta-analyses and systematic reviews have come to mixed conclusions regarding their use as well (e.g., Witte and Allen, 2000; Ruiter et al., 2014; Tannenbaum et al., 2015). In general, empirical data support that behavioral change is more likely when threat information is accompanied by high efficacy messaging (Witte and Allen, 2000; Ruiter et al., 2014; Tannenbaum et al., 2015), but this combination may still prove ineffective. Why? Unfortunately, the context and cues in which threat and efficacy are embedded into messages are sometimes at cross-purposes with the goal of behavior change.

From a cue-reactivity perspective, message designers can expect certain cues to trigger certain types of cognitive and motivational processing and behavior automatically due to incentive-sensitization (Robinson and Berridge, 1993; Carter and Tiffany, 1999). The incentive-sensitization model posits that repeated exposure to addictive substances and their cues can create “incentive-sensitization” in which individuals exhibit attentional biases and compulsive cravings when re-exposed to these cues (Robinson and Berridge, 1993; Carter and Tiffany, 1999). The influences of incentive-sensitization have been well documented in the substance addiction literature (Robinson and Berridge, 1993; Carter and Tiffany, 1999) as well as in work focusing on food cues, especially for the most palatable types of food (Sobik et al., 2005; Hou et al., 2011). With this previous research in mind, work in health communication has noted the appetitive and incentivizing effects of different types of substance cues (i.e., tobacco, vaping, alcohol, and food) in prevention messages, potentially creating unintended effects. These cues elicit approach tendencies indicated by increased craving, self-reported positivity, physiologically appetitive responses, and attention (Bailey, 2015, 2017; Clayton et al., 2017a,b, 2019a,b; Liu and Bailey, 2019; Sanders-Jackson et al., 2019) increased visual fixation (Sanders-Jackson et al., 2011); and greater memory for the cues (Clayton et al., 2017b; Bailey et al., 2018; Sanders-Jackson et al., 2019). The important take-away from this growing evidence is that message designers must understand how these cues function, particularly in fear appeal messaging, which use them to gain attention (Clayton et al., 2017a) and potentially inhibit message rejection (Bailey et al., 2018; Sarge and Gong, 2019). The studies presented here build on this previous work by considering social cue influences. Social facilitation of eating cues was examined across two experiments. Study 1 sought to determine whether social facilitation of eating cues induced more craving with simple visual still image stimuli. Study 2 then examined how the presence of these cues may influence effectiveness of more complex televised anti-obesity public service announcements (PSAs) containing fear appeals.

## STUDY 1

### Social Facilitation Cues

Social facilitation occurs when a behavior is increased due to the perceived presence of others (Zajonc, 1965; Cottrell et al., 1968; Bond and Titus, 1983; Baron, 1986). Some social facilitation studies find that mere presence of others is enough to trigger facilitation (Zajonc, 1965); others find individuals must recognize being watched for their behavior to be influenced (Cottrell et al., 1968). Suggested mechanisms include increased arousal (Zajonc, 1965), increased attention (Baron, 1986), self-presentational concerns (Bond and Titus, 1983), and evaluation apprehension (Cottrell et al., 1968). One behavior in which social facilitation research has been abundant is eating.

Previous research has indicated that social cues and settings influence how much food is consumed by individuals (De Castro, 1990; Clendenen et al., 1994; Herman, 2017). Human eating is generally social. Perceptions of “ideal” meals involve eating in other people’s company (Sobal and Nelson, 2003). Further, people tend to eat more with others (without high evaluative contexts in place) especially in larger groups (De Castro, 1990) and when eating sessions are longer (Herman, 2017). When evaluative contexts are in place, self-presentational norms may encourage individuals to match behavior of others or eat less when restriction seems to be socially appropriate; women seem to be especially susceptible to the latter, depending on the context, social companions, and types of norms displayed (Mori et al., 1987; Roth et al., 2001; Hermans et al., 2008; Young et al., 2009; Higgs and Thomas, 2016). Suggested mechanisms behind this phenomenon mirror that of social facilitation in general and range from increased positive affect and arousal, greater exposure to food cues, and social modeling (Herman, 2017). In other words, social eating contexts provide more and longer exposure to both food and eating cues, which both generally increase positive affect, arousal, and appetitive motivational activation, leading to greater food intake (unless normative expectations preclude that behavior).

These co-occurring appetitive cues (food and eating) create an interpretation problem for those intending to understand their individual influences. Because organisms need food to provide the energy and nutrients required to sustain their bodily functions, food stimuli are thought to be primary appetitive motivationally relevant stimuli (Bradley et al., 2001; Bailey, 2015). Empirical findings have demonstrated that exposure to food cues automatically elicit appetitive motivational responses (Sobik et al., 2005; Hou et al., 2011). Thus, disentangling responses to food cues and responses to social eating cues is quite difficult; viewing someone eating naturally includes food cues. But, does the social nature of more than one person eating further increase appetitive responses, as would be predicted if social facilitation effects occur?

Three recent studies (Liu and Bailey, 2020; Samson and Buijzen, 2020, 2021) have found that mediated eating cues can increase attention, positive emotion, and appetitive responses toward foods. Samson and Buijzen (2020) found that viewing individuals eating foods with hedonic expressions, compared to neutral expressions, increased positive responses toward healthy foods. Liu and Bailey (2020) found viewers paid more attention and reported more purchase intentions when viewing ads with multiple individuals present. Samson and Buijzen (2021) found increased attention and positive responses toward healthier foods in particular.

Thus, empirical data suggest that the *mediated* mere presence of others has the ability to facilitate behaviors leading to eating. However, these studies do not directly disentangle food cues from social eating cues. Thus, the first experiment reported here tested whether mediated social eating cues increased appetitive responses more than food cues alone.

### Methods

Participants (*N* = 61) were predominantly female (59%), young (*M*_age_ = 20.38, *SD*_age_ = 2.38), and predominantly Caucasian (49.2%) undergraduate students at a large public university in the United States. They completed a 2 (social eating cue: individual eating vs. social eating) x 3 (repetition of cue exemplar) within-subject experiment utilizing still images of food and eating cues as stimuli. *A priori* power analysis using the G^*^power program (Faul et al., 2009) indicated for an 0.80 power estimate, specifying a standard small effect (0.20), a 0.05 alpha level, and 0.5 correlation among repeated measures, a 2×3 *F*-test required a sample size of 42.

These participants completed the 60-min, Institutional Review Board-approved protocol individually. After informed consent was obtained in a lab setting, participants were seated approximately 7 feet from a high definition 42 Inch LCD screen with access to a computer keyboard. Participants viewed a series of images, one at a time, which varied in cue type in one of two orders. They reported their craving level for the food depicted in each image *via MediaLab* software (Jarvis, 2014). Social eating cues were manipulated in images by varying the number of individuals present: only one individual eating versus a group of people eating. In all images, individuals’ faces and facial expressions (in particular their eyes and mouths) were visible and expressions were emotionally neutral to positive (smiling, laughing, engaged in eating). Individuals in the images were all relatively young to match the sample of participants. A mix of gender and race/ethnicity cues were present. The foods being eaten were all highly energy dense items (e.g., potato chips and ice cream) likely to induce craving (Drewnowski, 1997) to increase variability in responses. Three images were used in each cue level to increase generalizability of craving results to a type of cue rather than a particular food, for a total of six images. Table 1 describes the exemplar images used in each type of image.

**Table 1:** Study 1 still image descriptions.

	Individual Eating Cue Images	Social Eating Cue Images
Exemplar 1	Medium shot of a young man eating a potato chip	Medium shot of a group of young women eating ice cream cones
Exemplar 2	Medium shot of a young man eating pizza	Medium shot of mixed gender group eating cookies
Exemplar 3	Medium shot of a young woman eating a cookie	Medium shot of mixed gender group eating pizza

Craving was measured using the 8-item Alcohol Urge Questionnaire (Bohn et al., 1995) often used in cue-reactivity research altered to refer to food: for example, “Eating __ would make things seem perfect right now.” Each item used a 5-point scale from 1 = *do not at all agree* to 5 = *strongly agree*. A craving index was created for each food image by averaging the responses to the eight items, *α* = 0.96. These data were collected as part of a larger study examining individual and polysubstance cue-induced craving. Other non-overlapping data from this series are published here [blinded].

### Results

The hypothesis predicted that the presence of more than one individual eating would evoke greater craving than one individual eating. In order to test this hypothesis, craving index data were submitted to a 2 (social eating cue: individual eating vs. social eating) × 3 (repetition of image) repeated measures ANOVA. The predicted social cue main effect was found, *F*(1,60) = 4.997, *p* = 0.029, $\eta_{p}^{2}$ = 0.077, such that when more than one individual was present (*M* = 3.28, *SE* = 0.159), craving was higher than when only one individual was present (*M* = 3.12, *SE* = 0.159).

### Discussion

These results indicated that the mediated mere presence of multiple people eating does create a small but significant increase in craving. This supports that the social nature of social eating cues increases appetitive activation over and above food cues alone. Further, this expected outcome is important in understanding influences of multiple mediated contexts, not least of which is anti-obesity messages. Substance cues are used in these types of prevention messages in order to gain and keep attention (Clayton et al., 2017a) and potentially inhibit message rejection when a fear appeal is present (Bailey et al., 2018; Sarge and Gong, 2019). The following study examines social facilitation of eating cues in anti-obesity fear appeals to determine whether they also create the problematic, unintended outcomes that other incentivized substance cues do (e.g., craving for and intended use of the problematic substance).

## Study 2

Given that more than one-third of US adults are now obese (Hales et al., 2020), obesity prevention messaging must evolve. These messages often include imagery of individuals eating junk food, likely because the imagery immediately captures attention and directly communicates behaviors to limit (Clayton et al., 2017b). Further, this imagery likely creates more positive evaluations of the messages overall because it is positively valent and appetitively motivating (Bailey, 2015; Bailey et al., 2018). This second reason may be even more likely when message designers are creating fear appeals, which are messages that communicate “the harmful physical or social consequences of failing to comply with message recommendations” (Hale and Dillard, 1995, p. 65).

Fear appeals are so-named because they rely on an audience associating experienced fear with certain behaviors. In this case, associating fear of health consequences such as heart disease and diabetes with eating junk food. However, the effectiveness of fear appeals is often questioned. In some cases, fear appeal messages can create stimulus rejection responses (e.g., Leshner et al., 2018) and in others, psychological reactance responses including anger and counterarguments (e.g., Nabi et al., 2008; Clayton et al., 2019a,b, 2020). One potential solution to this problem is for message designers to include enough positive information that the messages do not create rejection or reactance. This positive information often comes in the form of efficacy information (Witte and Allen, 2000; Nabi et al., 2008), based on theoretical assumptions and empirical data that support efficacy may trigger danger rather than a fear protection, creating message-aligned responses (Witte, 1994). This efficacy information may elicit specific discrete positive emotions (e.g., hope; Nabi and Myrick, 2019) that counter deleterious effects of fear. The positive information needed to inhibit stimulus rejection responses may also come in the form of positive emotional content (Bailey et al., 2018; Liu and Bailey, 2019; Sarge and Gong, 2019; Liu and Bailey, 2020); however, as discussed above, this may mean message designers inadvertently provide cues encouraging unintended responses (e.g., craving) if they select substance cues to fill this role.

As Bailey et al. (2018) noted, by including food items in anti-obesity fear appeals, the messages present cues that automatically elicit appetitive responses (Boysen et al., 1996; Bailey, 2015), which, in the broader context of a fear appeal message, creates messaging that is coactive, or containing both appetitive (positive) and aversive (negative) motivationally relevant content. Their findings indicated that fear appeals including food cues, especially in messages that were highly arousing, created memory decrements likely due to cognitive overload (Bailey et al., 2018), though these types of messages were rated as more engaging and likable. Similar work by Clayton et al. (2019b) demonstrated that these messages generate “motivational dissonance” in viewers as they rate the messages as both positive and negative. Thus, message designers may be seeing benefits of positive affect which, all else being equal, yield higher ratings of engagement and likability, and potentially greater perceived efficacy.

The Extended Parallel Process model (Witte, 1994) proposes that if a threat is perceived as severe, individuals will then assess their susceptibility to and efficacy in dealing with the threat. If individuals do not believe they are susceptible, they will not perceive the threat as relevant and may not move on to make efficacy assessments. Two types of efficacy are thought to be assessed: self-efficacy and response efficacy. Self-efficacy is the ability of the individual to deal with the threat, and response efficacy is the evaluation of whether the recommended action will actually lessen the threat (Witte, 1994). When both self and response efficacy are high, individuals are more likely to adopt recommended actions, but if either or both are not sufficiently high, message rejection or reactance may occur.

Fear appeals often use peers to tailor messages to indicate susceptibility to targeted groups. Further, fear appeals often contain highly threatening information to ensure that a threat is perceived as severe; but, in order to keep individuals from rejecting the information and recommendations, positive contents are used to ensure individuals experience greater self and response efficacy. Positive affect, induced by different positive emotional contents, has been shown not only to increase self and response efficacy (Guan and Monahan, 2017), but also create better attitudes toward the health behaviors being promoted in the messaging (Dillard and Peck, 2000). Previous research has also indicated that positive affect may increase intent to engage in a message’s recommended health behaviors (Previte et al., 2015). Therefore, positive content in health messages may increase recommended behaviors, all else being equal. However, some positive content may also elicit other responses.

### Social Facilitation Cues: Implications for Fear Appeal Outcomes

As discussed above, food-related cues create positive affect but also may encourage unhealthy eating behaviors (Bailey et al., 2018). Social eating portrayals may further exacerbate this due to social facilitation effects (De Castro, 1990; Clendenen et al., 1994; Drewett, 2007; Herman, 2017; Liu and Bailey, 2019; Samson and Buijzen, 2021), as replicated in Study 1.

Based on the large body of research indicating social facilitation of eating, predictions for emotion, attention, and behavioral outcomes are straightforward. However, the mere presence of others in fear appeals likely also has other influences *via* the assessment of social norms. Social norms are “rules and standards that are understood by members of a group, and that guide or constrain social behaviors” (Cialdini and Trost, 1998, p. 152). Descriptive social norms, in particular, deal with communicating prevalence of appropriate behaviors. Descriptive social norms may be interpreted as “if a lot of people are doing this, it’s probably a wise thing to do,” which serves to initiate norm-congruent behavior (Cialdini, 2007, p. 264). Thus, these norms are often communicated by observing others, implicitly functioning to influence behaviors, but also can be communicated explicitly (Hogg and Reid, 2006). Studies have suggested that social norms commonly influence eating, especially in contexts in which social comparisons and self-presentational concerns are important (Roth et al., 2001; Higgs and Thomas, 2016) In these cases, matching norms, in which one is expected to match the eating amounts and styles of others as well as the expectations of the situation can be prevalent, but a minimal eating norms can also be present, especially for women (Roth et al., 2001; Higgs and Thomas, 2016).

Social norm manipulations are often utilized in health campaigns to assess and correct misperceptions of how much peers engage in unhealthy (or do not engage in healthy) behaviors in order to encourage compliance with health recommendations (McAlaney et al., 2011; Dempsey et al., 2018). Some scholars have noted that social norms approaches may be particularly useful as a counterpoint to fear appeals due to their over-depiction of high-risk behaviors (McAlaney et al., 2011; Dempsey et al., 2018). For this reason, when fear appeals 1. contain highly threatening information to ensure that a threat is perceived as severe and 2. depict viewer peer groups demonstrating the risky behaviors in order to highlight viewer susceptibility, the descriptive norm implicitly communicated is that the behavior is risky but common.

These theoretical predictions and empirical findings suggest that when social eating cues are present in messages, they may have different behavioral influences than designers intend. Thus, these hypotheses are posed: portraying a group of people eating (versus one person eating) in obesity prevention PSAs will generate higher levels of (H1) positive affect, (H2) perceived threat severity and susceptibility, and (H3) self- and response efficacy, but (H4) create less intention to avoid the foods in question. Further, because these appetitive cues are embedded in aversive fear appeal messaging, their presence has implications for motivational activation and subsequent cognitive and emotional processing of the messages, including attention and arousal.

### Social Facilitation Cues: Implications for Motivational Coactivation

The motivated cognition framework argues that cognitive processing is biased toward information related to our biological imperative to survive and pass on genes (Bradley et al., 2001). Thus, information that is related to survival, threat avoidance, seeking of opportunities for food and mates, and other base-level drives is most automatically attention-grabbing and consequent for behavior. Theory predicts and empirical evidence has found that automatic behaviors are organized by activation in either or both of two motivational systems, the appetitive and the aversive (Cacioppo and Gardner, 1999; Bradley et al., 2001; Norris et al., 2010). The appetitive system activates automatically upon encountering positive stimuli, which functions to support approach behaviors. The aversive system automatically activates upon encountering negative stimuli, helping individuals respond quickly to potential threats.

Given that many health communication messages contain fear appeals layered with positive emotional contents, including substances cues and social norms reinforcement, several researchers have begun to explore the processing of so-called coactive messages, which are messages that elicit activations of both the appetitive and aversive systems, either simultaneously or sequentially (Wang et al., 2012; Lang et al., 2013; Keene and Lang, 2016; Clayton et al., 2017a; Bailey et al., 2018). Behavioral and neural evidence have revealed the pattern of processing, emotional experience, and behavior that occur when individuals are experiencing coactivity (see Norris et al., 2010), which indicates that some mutual inhibition between appetitive and aversive substrates may occur (Berntson and Cacioppo, 2008). This may account for the relatively consistent finding that during coactivation, individuals pay more attention but experience overall less physiological (Wang et al., 2012) and self-reported arousal (Lang et al., 2013).

Thus, we expect to see motivational coactivation in response to fear appeals that contain social facilitation of eating cues. In response to onset of social eating cues embedded in fear appeal anti-obesity messages, we expect (H5) viewers will exhibit an autonomic pattern consistent with greater motivational coactivation across time, greater deceleration in heart rate and lesser skin conductivity level, compared to responses elicited by individual eating cues.

These hypotheses were tested in a fully within-subject experiment in which participants viewed televised anti-obesity PSAs that varied in the number of people portrayed eating (one individual vs. a group). Dependent variables were self-reported affect, perceived susceptibility and severity, self-efficacy and response efficacy, behavioral intentions to avoid unhealthy foods, and psychophysiological indicators of cognitive resource allocation (heart rate) and arousal (skin conductance) during message exposure.

### Methods

Participants (*N* = 83) were predominantly young (*M*_age_ = 22.04, *SD* = 9.20) and female (60.2%) undergraduate students at a large public university in the United States. They completed a 2 (social eating cue: individual eating vs. social eating) × 2 (repetition of exemplar message) repeated measures within-subject experiment utilizing televised fear appeal anti-obesity messages as stimuli. For the physiological investigation, we wanted to examine the evoked autonomic responses to the eating cues. In order to do so, we selected six examples of eating cues within the messages: three that contained one person eating and three that contained multiple persons eating. Previous work examining the effects of emotionally social cues on self-reported affect including positivity, negativity, and arousal (Samson and Buijzen, 2020) indicated effect sizes ranging from 0.23–0.44. However, as Study 1 reported a lower effect size for craving self-reports, and psychophysiological studies also often report smaller effect sizes (as well as present higher correlations between repeated measurements), we specified a small effect size (0.15), an *α* of 0.05, and 0.6 correlation between repeated measurement in the G*Power program (Faul et al., 2009). The proposed 2×2 *F*-test of the self-reported data and 2×3 *F*-test of the physiological data require at least 72 and 60 participants, respectively, to achieve 0.80 power estimates.

These participants completed the IRB-approved protocol individually. After informed consent was obtained, participants were seated approximately 4 feet from a 42 Inch screen. The experimenter placed physiological data collection sensors while explaining the protocol. *MediaLab* software (Jarvis, 2014) was used to display each PSA. The same questionnaire was answered after each, randomized within-scale. When participants had viewed and rated all messages, they answered demographic questions and were debriefed, thanked, and dismissed. The entire procedure lasted approximately 60 min. These data were collected as part of a larger study examining emotional responses to fear appeals. Other data collected are published here (blinded).

### Stimuli Selection

#### Messages

Four 30-s televised anti-obesity fear appeal PSAs were selected to meet objective criteria: the messages had to contain information that communicated “harmful physical or social consequences” of obesity (Hale and Dillard, 1995, p. 65) such as risk of disease or death, and all messages had to contain food cues for items the message indicated were unhealthy. And lastly, individuals in the PSAs had to be consuming these unhealthy foods. These PSAs were grouped into two types to satisfy the objective manipulation of how many individuals were eating in the PSA: 1 individual or more than 1. Two messages of each type were used in order to be better able to generalize the findings to a type of social cue rather than a singular message. The messages varied in terms of content in other ways (e.g., gender, age, race/ethnicity of those depicted, and contexts). Facial expressions were generally visible in all messages. Emotional expressions ranged from neutral to positive (smiling, engaged in eating) to more negative (frowning) during expression of health risks. Table 2 lists brief descriptions of each exemplar message.

**Table 2:** Study 2 video PSA descriptions.

	Individual Eating Cue Messages	Social Eating Cue Messages
Exemplar 1	Televised PSA depicting an adult male eating unhealthy heavily processed and calorie-dense foods from a vending machine on a work break with a commentary about the health risks of doing so, including obesity and heart disease	Televised PSA depicting four family members (presumably a mother, father, son, and daughter) eating a fast food meal of burgers and chips/fries around a dinner table with a commentary indicating eating fast food may cause a fast death
Exemplar 2	Televised PSA depicting a mother giving her child a fast food burger with a comparison made of giving the child other addictive unhealthy substances.	Televised PSA depicting an adult male eating unhealthy heavily processed and calorie-dense foods with others across multiple meals juxtaposed with later portions of the message depicting health consequences of those eating choices including obesity

#### Eating Cues

Within the messages that were identified as containing individual and social eating cues, three exemplar onsets of eating cues were selected. The cues met these criteria: they contained eating cues, they were changes from frames that did not contain eating cues to frames that contained eating cues, they were at least 6 sec from the other selected exemplar cues.

### Dependent Variables

#### Self-Reported Emotional Experience

Self-reported emotional experience was measured using 3 items: 1. “Overall, how positive/pleasant/happy did the message make you feel?”; 2. “Overall, how negative/unpleasant/unhappy did the message make you feel?”; 3. “Overall, how aroused/excited/awake did the message make you feel?” Response options ranged from *Not at all* (1) to *Extremely* (7). Here, we followed Cacioppo and Berntson (1994), who suggest emotional responses should be assessed in underlying dimensions. Thus, each component was analyzed separately, not as an index.

#### Perceived Severity

Perceived severity of the threat was assessed with 3 items: “Based on this ad, I believe that obesity is serious” and “Based on this ad, I believe that obesity is severe” and “Based on this ad, I believe that obesity is significant.” Response options ranged from *strongly disagree* (1) to *strongly agree* (7). The inter-item consistency was acceptable with a Cronbach alpha of 0.88.

#### Perceived Susceptibility

Perceived susceptibility to the threat was assessed with 3 items: “Based on this ad, it is possible that I would suffer from obesity” and “Based on this ad, I at a risk for becoming obese” and “Based on this ad, it is likely that I will be obese.” Response options ranged from *strongly disagree* (1) to *strongly agree* (7). The inter-item consistency was acceptable with a Cronbach alpha of. 91.

#### Perceived Self-Efficacy

Perceived self-efficacy was assessed using 3 statements based on Witte et al. (1996): “I am able to use the recommendation(s) provided in this video to prevent obesity” and “The recommendation(s) is/are easy to do to prevent obesity” and “The recommendation(s) to prevent obesity is/are convenient.” Response options ranged from *strongly disagree* (1) to *strongly agree* (7). The inter-item consistency was acceptable with a Cronbach alpha of 0.81.

#### Perceived Response Efficacy

Perceived response efficacy was assessed with 3 statements based on Witte et al. (1996): “The recommendation(s) is/are effective in preventing obesity” and “If I follow the recommendation(s), I am less likely to become obese” and “The recommendations work for preventing obesity.” Response options ranged from *strongly disagree* (1) to *strongly agree* (7). The inter-item consistency was acceptable with a Cronbach alpha of 0.77.

#### Behavioral Intention

Behavioral intention was assessed with 3 items adapted from Lee and Bichard (2006): “I would like to follow the recommendations made, such as eating healthy food” and “I am planning to change my unhealthy eating and drinking habits very soon” and “I do not plan to ever change my unhealthy eating and drinking behaviors unless I see my health suffering” (Reverse coded). Response options ranged from *strongly disagree* (1) to *strongly agree* (7). Inter-item consistency was reasonable with a Cronbach alpha of 0.61.

#### Heart Rate

Heart rate data were collected to index cognitive resource allocation across time. Heart rate deceleration is commonly used in media message research as an overtime, unobtrusive indicator of cognitive resource allocation to encoding stimuli. An assumption of psychophysiological measures is that the work of the body is more influential on physiological systems than the work of the brain, but both are continuously and simultaneously influential on outcomes like autonomic activity, and consequently, heart rate and other physiological metrics. A great many studies have found that heart rate deceleration is indicative of greater external stimulus processing, even when messages are arousing in nature, while acceleration is indicative of internal mental focus, external stimulus rejection, imagery creation, and preparation for action, depending on context (see Lang, 1994; Lang et al., 2009; Potter and Bolls, 2012 for reviews). A Biopac MP-150 wireless amplifier and two disposable 8 mm Ag-AgCI electrodes placed on the forearms with a ground on the non-dominant wrist were used to collect Raw electrocardiogram data. Raw data were sampled at 1000hz and cleaned off-line with Biopac Acqknowledge software. Recording artifacts were identified and corrected using interpolation. Average beats per minute (BPM) per second data were computed for each second of exposure for each participant.

#### Skin Conductivity Level

Skin conductivity level (SCL) data were collected to index sympathetic arousal (Potter and Bolls, 2012). Tonic SCL was recorded using a Biopac MP150 wireless unit that passed a constant measurement voltage of 0.5v between two disposable 8 mm Ag/AgCl electrodes on the non-dominant hand. Raw data were sampled at 1000hz. Average SCL data were computed for each second of exposure for each participant.

### Data Treatment and Analysis

Self-report data were submitted to a 2 (social eating cue: individual eating vs. social eating) × 2 (repetition) repeated measures ANOVA. Prior to analysis, the physiological data (heart rate and skin conductivity level) were transformed to assess change from onset of eating cues (see information regarding selection of eating cue exemplars above). The first second of each cue onset was identified, and the values for these onsets in the average BPM and average SCL data were located. The values of the 5 sec following each cue onset were also located. The value of the first second of onset was used as a reference point and subtracted from each of the following five values to construct a change from onset of eating cue trajectory of BPM and average SCL data. This was done in order to better examine the evoked responses to the cues and better isolate the changes in these two metrics due to the cues themselves rather than the many factors that play a role in fluctuations of heart rate and skin conductivity (see Potter and Bolls, 2012 for a discussion of this data treatment method). These change-transformed data were submitted to a 2 (social eating cue: individual eating vs. social eating) × 3 (repetition of exemplar) × 6 (time in seconds from onset of the eating cues) repeated measures ANOVA. In order to deal with the autocorrelated nature of the physiological data, which violated sphericity assumptions, Hyun–Feldt corrections were utilized. Original and corrected degrees of freedom are reported.

### Results

#### Emotional Responses

**Figure 1:**
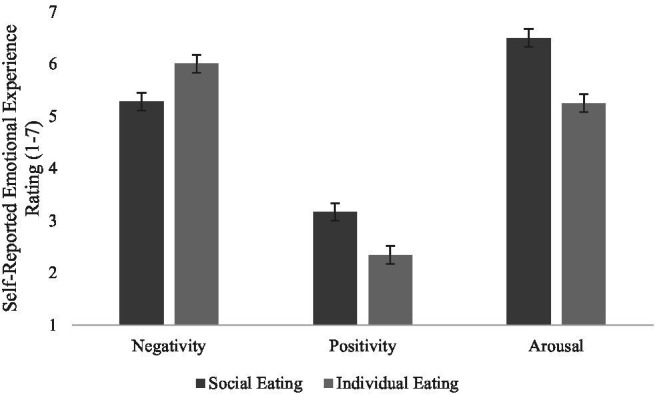
Self-reported emotional experience as a function of type of social eating cues present in the obesity PSA messages.

Hypothesis 1 predicted that a group of people portrayed eating would increase positive affect compared to an individual. The predicted social eating cue main effects on positive affect, *F*(1,82) = 26.99, *p* < 0.001, $\eta_{p}^{2}$ = 0.25, negative affect, *F*(1,82) =13.73, *p* < 0.001, $\eta_{p}^{2}$ = 0.14, and emotional arousal ratings, *F*(1,82) = 67.12, *p* < 0.001, $\eta_{p}^{2}$ = 0.45, were found. As can be seen in Figure 1, when social eating cues were present, viewers rated messages as less negative, more positive, and more emotionally arousing. Hypothesis 1 was supported.

#### Perceived Severity and Susceptibility

**Figure 2:**
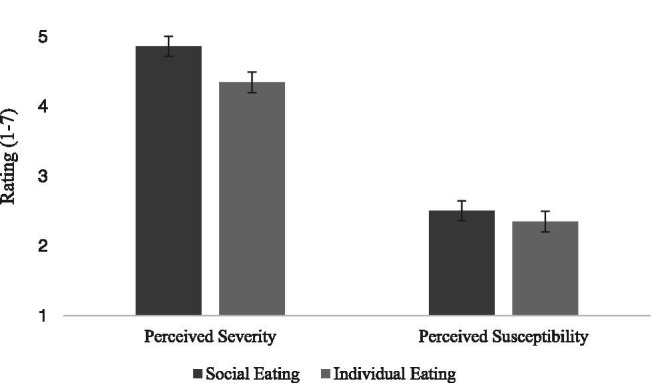
Perceived severity of and susceptibility to threat ratings as a function of type of social eating cues present in the obesity PSA messages.

Hypothesis 2 predicted that more people portrayed eating would increase perceived threat severity and susceptibility compared to individuals eating. This predicted main effect of social eating cue was found on perceived severity, *F*(1,82) = 22.30, *p* < 0.001, $\eta_{p}^{2}$ = 0.22, but not on perceived susceptibility, *F*(1,82) = 2.03, *p* = 0.14, $\eta_{p}^{2}$ = 0.03. As can be seen in Figure 2, when social eating cues were present, viewers reported more perceived severity and more perceived susceptibility (though, again, this latter difference was not statistically significant). Hypothesis 2 was partially supported.

#### Perceived Efficacy

**Figure 3:**
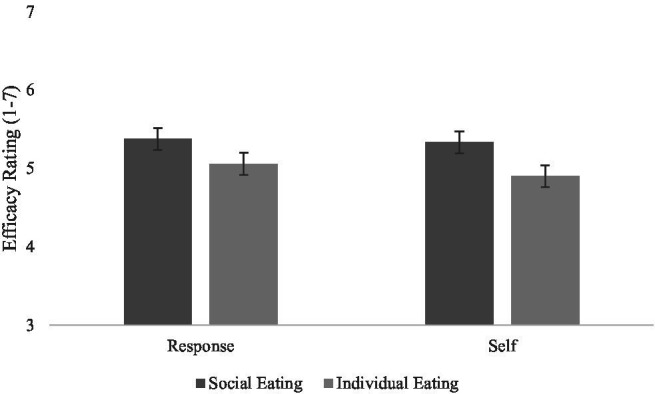
Efficacy ratings as a function of type of social eating cues present in the obesity PSA messages.

Hypothesis 3 predicted that more people portrayed eating would increase response and self-efficacy compared to individuals eating. This predicted main effect of social eating cue was found on response efficacy, *F*(1,82) = 7.54, *p* = 0.007, $\eta_{p}^{2}$ = 0.08, and self-efficacy ratings, *F*(1,82) = 11.53, *p* = 0.001, $\eta_{p}^{2}$ = 0.12. As can be seen in Figure 3, when social eating cues were present in PSAs, viewers reported more response efficacy and self-efficacy. Hypothesis 3 was supported.

#### Behavioral Intentions

Hypothesis 4 predicted that a group of people portrayed eating would decrease healthy eating intentions compared to an individual portrayed eating. The main effect of social eating cue was found on eating intention ratings, *F* (1,82) = 26.00, *p* = 0.001, $\eta_{p}^{2}$ = 0.24. When social eating cues were present, viewers reported fewer intentions to change their unhealthy eating behaviors (*M* = 2.62, *SE* = 0.10) compared to when individual eating cues were present (*M* = 3.29, *SE* = 0.12). Hypothesis 4 was supported.

#### Motivational Coactivation

**Figure 4:**
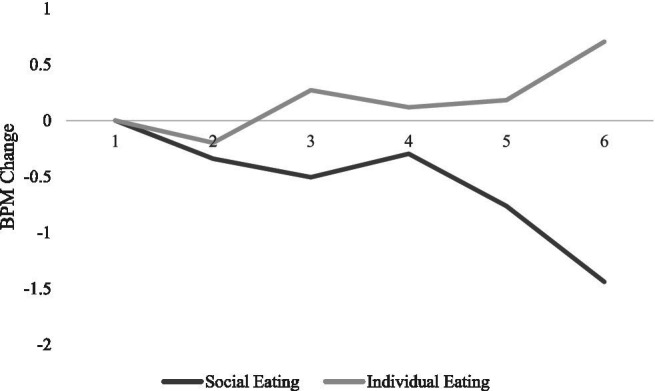
Heart rate in beats per minute as a function of time of exposure and type of social eating cues present in the obesity PSA messages.

**Figure 5:**
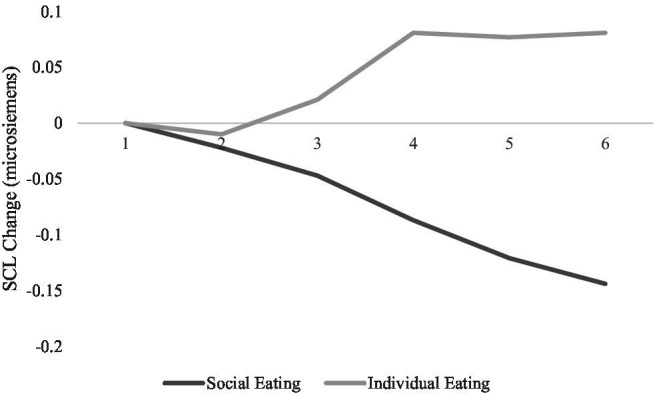
Skin conductivity level as a function of time of exposure and type of social eating cues present in the obesity PSA messages.

Hypothesis 5 predicted that when social eating cues were presented in fear appeal messages, individuals would exhibit an autonomic pattern consistent with coactivation across time, more deceleration in heart rate and lesser skin conductivity level. The predicted interactions of social eating cue with time were found on the heart rate data, *F*(5,320/1.9,122.5) =6.87, *p* < 0.001, $\eta_{p}^{2}$ = 0.10 and SCL data, *F*(5,320/1.4,90.7) = 8.19, *p* = 0.002, $\eta_{p}^{2}$ = 0.11, such that when a group of people were portrayed eating, viewers exhibited more deceleration in heart rate and less SCL overall. See Figures 4, 5. Hypothesis 5 was supported.

### Discussion

This study examined the influence of social eating cues in anti-obesity fear appeals on individuals’ reported emotional experiences, perceived severity and susceptibility, efficacy ratings, healthy eating intentions, and motivational coactivation indexed *via* autonomic patterns in heart rate and skin conductivity. Results indicated that the messages containing a group of individuals eating created not only more positive emotional responses, but also more response and self-efficacy. Further, the social facilitation components functioned to increase perceived threat. However, the social eating imagery elicited lower ratings of healthy eating intentions, as predicted.

Perceived susceptibility was not significantly affected by social eating cues. Group affiliations with the persons portrayed in the messages may not have been strong enough to induce this effect. Further, the mixture of social norms portrayals of unhealthy eating paired with severe threat information may have made the outcomes described in the message (obesity and obesity-related illness) seem unlikely, yielding lower perceptions of susceptibility (see McAlaney et al., 2011 for related discussion).

## General Discussion

The findings presented here are in line with previous health communication and cue-reactivity research, but may be counterintuitive for health communication message designers. Food and eating cues in real and mediated contexts automatically elicit appetitive motivational activation (De Castro, 1990; Clendenen et al., 1994; Bailey, 2015, 2017; Herman, 2017; Liu and Bailey, 2020), which creates more positive affect that yields greater response and self-efficacy (Previte et al., 2015; Guan and Monahan, 2017). However, this automatic appetitive motivation is concurrently activating approach and consumption behaviors, leading to fewer intentions to actually change unhealthy eating behaviors. Further, the psychophysiological data presented here support that social eating cues are experienced coactively when embedded in fear appeals. This confirms previous explanations regarding why message designers may turn intuitively to social eating cues. The motivational coactivation elicited by social eating cues in fear appeals facilitates attention to the messages as well as overall positive affect and message evaluation. Thus, even though televised fear appeal PSAs with social eating imagery intend to persuade people not to consume unhealthy food, the increased appetitive motivational responses generated may create unintended and opposite behavioral consequences from those encouraged by the messaging.

Overall, these data tell an interesting story about the counterproductive effects of the presentation of food-related cues in anti-obesity PSAs that may be discouraging positive behavior changes. These results may seem paradoxical but align well with a social norms approach as well as a cue-reactivity point of view. Eating is an inherently social behavior in humans, and the indications of eating encourage eating in others (de Castro, 1990). Because these kinds of eating and food-related cues are primary biologically motivators, creating positive affect, which in turn makes individuals feel more positive and efficacious while simultaneously encouraging them to eat (Bailey, 2015). Taken together, these findings indicate that food-related cues, especially those that also engage social facilitation, play a role in appetitive motivation and reward and subsequently, cognitive and emotional processing. It is therefore important for message designers to avoid presenting food-related cues in obesity prevention messages if the goal is to discourage overeating or eating specific foods.

In sum, the current study examines potential detrimental effects of social eating cues in PSAs. While prior information tailoring studies have mainly focused on how individual and social factors moderate the effects of health message frames, this study was conducted to examine cue-elicited responses when watching obesity prevention fear appeals. Our results suggest that those obesity prevention PSAs, which were designed to promote healthy eating behaviors, might have counterproductive effects when including social eating cues. Therefore, this study contributes to existing literature on tailoring health interventions by suggesting that similarity and liking cues may be facilitated by more general social cues. Further, this study provides practical suggestions for message designers and health professionals. If message designers intend to grab attention and soften fear appeals with positive affect-inducing stimuli, social eating cues are not the best choice.

Although the current study has provided some insight into how social eating cues in anti-obesity PSAs influence individuals’ emotional experiences, perceived threat, efficacy ratings, and health intentions, limitations should be considered. First, self-reported emotional and behavioral responses were used as outcome variables, which may be subject to social desirability biases. Future studies should use measures less susceptible to these biases, including actual eating behavior. Second, we utilized a convenience sample of college students. While useful for looking at the impacts of social eating cues in this within-subject experimental design, this sample’s response pattern may not be generalizable to other groups. Next, though this study did utilize multiple exemplars within each eating cue category across both studies, they were limited to two or three exemplars each. Future work should replicate and expand the number of exemplars to a larger pool of exemplars to better defend against case-category confounds (see O’Keefe, 2015). Lastly, while this study examined the effects of social eating cues on emotional responses, health intentions, and self-efficacy and response efficacy, there may be other factors that also influence the impacts of social eating cues in PSAs. First, this study did not consider reactance, though many previous studies support that reactance is a crucial factor in fear appeal processing (e.g., Clayton et al., 2019b). Secondly, we did not consider weight status of participants, though some data indicate weight status may play a role in anti-obesity message effectiveness (Shentow-Bewsh et al., 2016; So and Alam, 2019). Further, many individual differences may moderate emotional and behavioral responses in the social context of eating. For example, gender seems to be an important factor in social eating scenarios (Mori et al., 1987; Hermans et al., 2008; Young et al., 2009), especially in combination with weight status and diet restriction (De Luca and Spigelman, 1979; Herman et al., 2003). Women are more likely to respond to social eating cues, either eating more or less, depending on the context, social companions, and social norms displayed. While this study was designed to minimize the influence of individual differences by comparing the influence of social eating cues within individual, future work should examine the interaction of these noted and other individual differences with social eating cues in influencing health prevention message effectiveness.

Despite these limitations, these findings contribute to knowledge regarding health messaging, especially in biologically relevant contexts such as discouraging unhealthy eating. Additional research considering how cues that create automatic biological responses are altering the intended effects of health messaging is necessary.

## Data Availability Statement

The raw data supporting the conclusions of this article will be made available by the authors, without undue reservation.

## Ethics Statement

The studies involving human participants were reviewed and approved by Florida State University Institutional Review Board. The patients/participants provided their written informed consent to participate in this study.

## Author Contributions

RB: writing, experimental design, stimulus, and data cleaning and analysis. TW: writing, experimental design, stimulus, and data collection and cleaning. JL: writing, experimental design, stimulus, and data cleaning. RC: writing, experimental design, and data collection. KK, VD, and FK: writing. All authors contributed to the article and approved the submitted version.

## Conflict of Interest

The authors declare that the research was conducted in the absence of any commercial or financial relationships that could be construed as a potential conflict of interest.

## Publisher’s Note

All claims expressed in this article are solely those of the authors and do not necessarily represent those of their affiliated organizations, or those of the publisher, the editors and the reviewers. Any product that may be evaluated in this article, or claim that may be made by its manufacturer, is not guaranteed or endorsed by the publisher.
